# Facteurs associés à l’hésitation vaccinale en Afrique au sud du Sahara: revue de la literature

**DOI:** 10.11604/pamj.2026.53.143.49923

**Published:** 2026-03-26

**Authors:** Amadou Tila Kebe, Bakary Diarra, Issa Kalossi, Bocar Mahamane Traore, Léon Savadogo, Hamadoun Sangho

**Affiliations:** 1 Département d'Enseignement et de Recherche en Santé Publique et Spécialités, Faculté de Médecine et d'Odontostomatologie, Université des Sciences, des Techniques et des Technologies de Bamako, Bamako, Mali,; 2 Institut National de la Santé Publique, Bamako, Mali,; 3 Direction Régionale de Santé de Tombouctou, Tombouctou, Mali,; 4 Université Senghor, Alexandrie, Égypte

**Keywords:** Refus de vaccination, facteurs associés, hésitation vaccinale, Afrique subsaharienne, réticence, COVID-19, HPV, Vaccine refusal, associated factors, vaccine hesitancy, sub-Saharan Africa, reluctance, COVID-19, HPV

## Abstract

La vaccination est une intervention clé pour réduire la morbidité et la mortalité infanto-juvéniles. Son succès repose essentiellement sur son acceptation par les populations. Cette étude vise à comprendre les déterminants de l'hésitation vaccinale en Afrique au sud du Sahara. La démarche méthodologique a consisté en une revue systématique de la documentation, réalisée à travers la consultation de PubMed et de la littérature grise, selon les critères Population, Intervention, Comparison and Outcome (PICO) et les recommandations PRISMA. Sur 32 études incluses, 28 étaient évaluées par les pairs, tandis que les autres provenaient de mémoires et de thèses des étudiants en master et des doctorants en sciences de la santé. Les principaux facteurs d'hésitation incluent l'âge jeune, le milieu rural, le faible niveau d'instruction, les doutes sur la sécurité ainsi que l'efficacité des vaccins, mais aussi la crainte d'éventuels effets secondaires. La méfiance envers les gouvernements et l'influence des médias sociaux jouent également un rôle. Cette revue permet d'identifier ces barrières et de proposer des solutions pour améliorer l'adhésion aux vaccins, notamment ceux du Programme Élargi de Vaccination et ceux contre la COVID-19.

## Abstract (en)

Vaccination is a key intervention for reducing infant and child morbidity and mortality. Its success depends largely on public acceptance. This study aims to understand the determinants of vaccine hesitancy in sub-Saharan Africa. The methodological approach consisted of a systematic review of the literature, conducted through consultation of PubMed and grey literature, according to Population/Patient, Intervention, Comparison, and Outcome (PICO) criteria and Preferred Reporting Items for Systematic Reviews and Meta-Analyses (PRISMA) recommendations. Of the 32 studies included, 28 were peer-reviewed, while the others were taken from dissertations and theses by master's and doctoral students in health sciences. The main factors contributing to hesitancy include young age, rural location, low educational attainment, doubts about vaccines safety and efficacy and fear of possible side effects. Mistrust of governments and the influence of social media also play a role. This review identifies these barriers and proposes solutions to improve vaccine uptake, particularly for the Expanded Programme on Immunisation and COVID-19 vaccines.

## Introduction

Bien qu'elle figure parmi les interventions reconnues en matière de prévention comme les plus efficaces, la vaccination est de plus en plus perçue par une proportion croissante de la population comme un risque. Selon Eve Dubé *et al*. en 2013, le manque de confiance dans les vaccins est désormais considéré comme une menace pour le succès des programmes de vaccination [1]. L'hésitation vaccinale contribuerait en partie à la baisse de la couverture vaccinale, à l'augmentation du risque d'apparition de maladies évitables par la vaccination et d'épidémies [1]. L'Organisation mondiale de la Santé (OMS) l'a classée parmi les 10 menaces majeures pour la santé mondiale [2]. Tant du point de vue individuel que celui de la santé publique, il est préférable et plus rentable d'adopter une approche préventive plutôt que curative [2]. D'après Anne Marie Moulin, les programmes de vaccination universelle et élargie sont d'une importance capitale protégeant la majorité des enfants à travers le monde contre des maladies infectieuses qui entraînaient autrefois des millions de décès [3]. Selon l'OMS, chaque année, la vaccination permet d'éviter plus de 2,5 millions de décès parmi les enfants âgés de moins de 5 ans à l'échelle mondiale (OMS, 2011) [4].

En 1974, l'OMS a instauré le Programme élargi de vaccination (PEV) afin d'assurer que tous les enfants aient accès à 4 vaccins systématiquement recommandés [5]. D'après le rapport sur les 10 grandes réalisations de la santé publique des *Centers for Disease Control and Prevention (CDC)*, des progrès notables ont été réalisés dans le développement de vaccins supplémentaires et des vaccins approuvés sont désormais disponibles pour prévenir 25 maladies en Afrique [4]. Larson *et al*. en 2015 ont mené l'une des plus grandes études réalisées sur toutes les régions de l'OMS, impliquant 67 pays, mais n'incluant malheureusement que 6 pays africains de la zone OMS Afro, notamment l'Algérie, l'Afrique du Sud, le Nigeria, la RD Congo, l'Éthiopie et le Ghana [6]. Leur étude montre que la zone OMS AFRO affiche une forte confiance dans les vaccins en termes d'efficacité, de sécurité et de compatibilité religieuse, bien que 2 à 9% expriment des désaccords [6]. Dans une étude récente dirigée par Gagneux-Brunon *et al*. diverses controverses et polémiques liées à la vaccination en Afrique ont amené certaines personnes à retarder ou refuser les vaccins recommandés, malgré leur accessibilité [7]. Cette réticence a accru la vulnérabilité des communautés face aux maladies infectieuses, déclenchant ainsi plusieurs flambées de maladies infectieuses à potentiel épidémique et évitables par la vaccination. Un exemple frappant, mentionné par Omer et al. dans leur article publié en 2009, est le boycott du vaccin contre la poliomyélite au Nigéria entre 2003 et 2004 [8]. Motivé par des rumeurs et la méfiance, ce boycott a entraîné une augmentation de l'incidence de la poliomyélite dans le pays entre 2002 et 2006, ainsi que des flambées sur trois continents [9].

Selon Jacquemot, l'OMS souligne dans son rapport qu’en Afrique, la disponibilité des vaccins n'est plus le seul obstacle à une couverture vaccinale efficace [10]. Ses recherches au Cameroun et au Sénégal indiquent que l'hésitation vaccinale est exacerbée par une prolifération de fausses informations, propagées notamment lors de périodes de pénurie et d'interruptions d'approvisionnement, ce qui accroît la méfiance [10]. Au Mali, une étude transversale descriptive menée à Gao par Kebe *et al*. en 2021, auprès de parents d'enfants et gardiens de moins de cinq ans [11]. Cette étude a révélé que 9,11% des parents ou responsables d'enfants étaient hésitants à vacciner leurs enfants [11]. En 2022, selon une étude de l'INSP (Institut national de la santé publique) du Mali sur les déterminants psychosociaux de la vaccination contre la COVID-19 [11], 32% ont été influencés par les professionnels de la santé, 24,5% par les proches et 32,2% par les médias par rapport au refus de vaccination [12].

Malgré une augmentation des couvertures vaccinales au fil du temps, les retards et refus vaccinaux continuent de contribuer à la morbidité et à la mortalité des enfants de moins de cinq ans en Afrique subsaharienne [13]. Certains auteurs ont réalisé des revues de la littérature qui ont été générales et très larges, qui n'ont pas assez pris en compte les spécificités de l'Afrique au sud du Sahara. Notre revue a révélé qu'il n'y avait pas de cadre conceptuel formel pour étudier le phénomène en Afrique au sud du Sahara. Une grande disparité existe entre les différentes zones de l'Afrique en termes de facteurs associés, déterminants et attitudes des parents face à la vaccination. Une grande partie des études au sud du continent africain a permis d'identifier les facteurs associés les plus importants. L'objectif général de cette revue de littérature est de rassembler, organiser et analyser les données existantes concernant l'hésitation vaccinale en Afrique au sud du Sahara et les principaux groupes de facteurs qui y sont associés. Plus spécifiquement, il s'agissait pour nous de voir l'ampleur de l'hésitation vaccinale, de déterminer les groupes de déterminants et aussi l'hésitation selon les types de vaccins.

## Méthodes

Cette revue systématique a été effectuée en suivant les directives et recommandations de PRISMA.

**Critères d'éligibilité:** nous avons utilisé les critères PICO pour formuler la question de recherche et les critères d'inclusion et d'exclusion des études [14].

**Critères d'inclusion:** les études quantitatives ou qualitatives sur l'acceptation, l'hésitation ou le refus de la vaccination réalisées en Afrique subsaharienne, ciblant la population générale, les agents de santé, les étudiants, les parents ou tuteurs d'enfants et publiées entre le 1^er^ janvier 2020 et le 31 mars 2025 ont été incluses dans l'étude. Cette période correspond à une durée suffisamment longue de publications (3 ans et demi) et permet d'identifier les recherches récentes (après 2020), tout en prenant en compte la période après la COVID-19 (après 2021).

**Critères d'exclusion:** certaines études ont été exclues pour les critères suivants: *accessibilité en ligne:* les études qui ne sont pas accessibles en ligne, comme des articles et des thèses non publiées en ligne. *Contexte de l'étude:* les études menées dans des contextes autres que les communautés ou les ménages, comme les études réalisées uniquement en milieu hospitalier ou dans des établissements de santé. *Géographie:* les études portant sur des populations en dehors de l'Afrique subsaharienne et les études ne spécifiant pas clairement le contexte géographique. *Variables d'intérêt:* les études qui ne traitent pas spécifiquement de l'hésitation vaccinale ou de la réticence à la vaccination, ou qui n'abordent pas les aspects des connaissances, pratiques, attitudes, et croyances liées à la vaccination. *Langue de publication:* les études publiées dans une langue autre que l'anglais ou le français. Ces critères d'exclusion ont permis de s'assurer que les études incluses sont pertinentes pour notre question de recherche et que les résultats obtenus sont fiables et comparables.

**Source d'informations:** la recherche a été effectuée sur les bases de données PubMed et Google Scholar. Les études et publications revues par les pairs, thèses d'exercice et de recherche ont été prises en compte. La recherche documentaire et bibliographique a été effectuée du 10 au 20 juillet 2025.

**Stratégies de recherche:** le moteur de recherche des données scientifiques PubMed a été principalement utilisé pour sélectionner les études et publications revues par les pairs sur l'hésitation vaccinale. La recherche a été effectuée à partir de l'équation de recherche suivante: « (“vaccine hesitancy” OR “vaccination hesitancy” OR “vaccine perception” OR “vaccination refusal” OR “vaccine hesitance” OR “vaccine reluctance” OR “vaccine acceptance” OR “vaccination acceptance” OR “vaccination acceptability” OR “vaccine intention”) AND ([“Health workers*”> “Health workers*”] OR [“parents*”“parents*”] OR [“community” AND “parents*”>“community” AND “parents*”]) ». Les articles ont été sélectionnés par la présence des mots-clés dans le titre, dans le résumé ou dans le corps de l'article. Les articles devaient être rédigés en anglais ou en français. Pour compléter l'examen de l'hésitation vaccinale en Afrique subsaharienne, on a aussi eu recours au site de l'OMS, à Google Scholar ainsi qu'aux thèses et mémoires soutenus devant un jury à partir d'octobre 2024.

**Sélection des études:** après avoir éliminé les doublons, les titres et résumés ont été examinés. Les articles retenus ont fait l'objet d'une lecture complète et ont été inclus sur la base des critères d'éligibilité. Les lectures ont principalement été menées par l'auteur principal. Les études éligibles étaient des études observationnelles, descriptives et/ou analytiques, évaluant l'hésitation vaccinale et/ou les facteurs associés à l'hésitation vaccinale chez les parents/tuteurs d'enfants et chez les agents de santé.

**Extraction des données:** on a rassemblé et examiné les informations relatives au titre, aux auteurs, à la date de recherche et de publication de l'étude, au pays d'origine, au genre d'étude et à la méthode de collecte des données. De plus, on a noté la nature des participants, le volume de l'échantillon ainsi que les résultats (taux d'hésitation vaccinale, taux d'acceptation, motifs de l'hésitation). Un tableau récapitulatif des informations clés a permis d'extraire les principales données susmentionnées. Une analyse thématique a été appliquée aux données extraites, et les résultats sont présentés sous forme de tableaux et de schémas.

## Résultats

**Figure 1:**
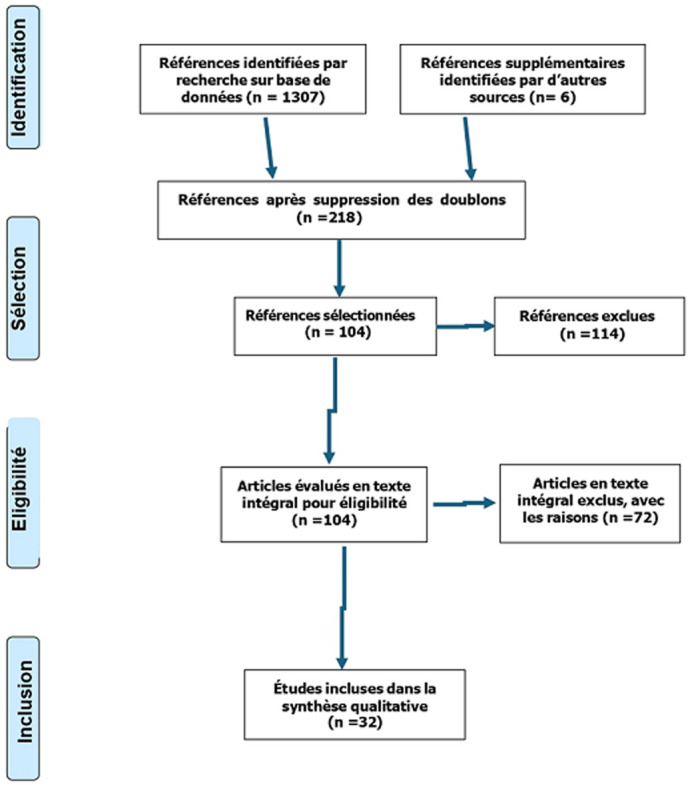
diagramme de flux de la sélection des études à travers l'approche PRISMA

À partir d'un total de 1313 études identifiées (à la suite de la revue initiale et actualisée de la littérature dans les bases de données sélectionnées), nous avons examiné 218 études pour déterminer leur admissibilité selon les critères de recherche et supprimé 1095 qui sont soit des doublons soit d'autres critères de non-inclusions. Sur les 218 restants, nous avons exclu 114 selon les critères d'exclusion cités plus haut. Nous avons donc finalement inclus un total de 32 études/rapports éligibles qui mentionnaient l'hésitation vaccinale comme résultat pour cette revue systématique (Figure 1).

**Caractéristiques des articles inclus dans cette revue:** au total, trente-deux articles ont été inclus dans cette revue pour analyse. Elles ont toutes été menées en Afrique. Douze (12) études ont été réalisées dans plusieurs pays (multicentriques). Les dates de publication des études s'étendent du 1^er^ janvier 2020 au 31 mars 2025. Exceptionnellement une étude publiée en février 2015 a été prise en compte pour la qualité des données qu'elle présente. Une analyse approfondie des études montre que les 32 enquêtes ont été réalisées dans 14 pays: Mali (2), RDC (2), Nigéria (3), Ghana (1), Éthiopie (2), Cameroun (2), Guinée (1), Sénégal (1), Malawi (1), Somalie (1), Afrique du Sud (4) et les 12 autres études sont multicentriques réunissant plusieurs pays d'Afrique au sud du Sahara. La majorité des articles étaient des études transversales (17), suivie d'expériences de revues systématiques (8) et d'analyses documentaires/rapports (7).

**Figure 2:**
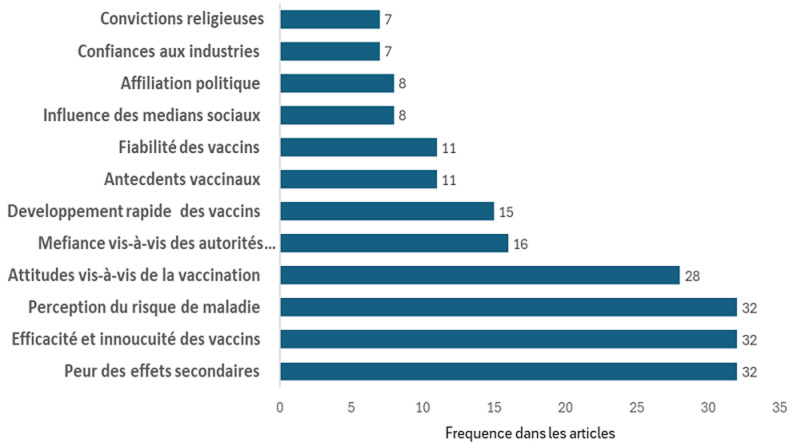
principales raisons d'hésitation vaccinale à partir des 32 études

**Les principales raisons d'hésitation à la vaccination:** une synthèse et analyse globale des différentes raisons de l'hésitation vaccinale montrent que la peur des effets secondaires, les préoccupations relatives à l'innocuité et l'efficacité vaccinale ainsi que la perception du risque sont les raisons les plus citées (32 fois), suivies des attitudes vis-à-vis de la vaccination (28 fois) et de la méfiance vis-à-vis de la vaccination dans 16 des articles analysés. Le développement rapide des vaccins, qui est souvent associé aux théories complotistes, est cité 15 fois, tandis qu'à la fois les antécédents vaccinaux et la fiabilité des vaccins ne sont notés que 11 fois. Le dernier groupe de raisons réunit l'influence des médias, l'affiliation politique ainsi que la confiance dans les industries pharmaceutiques et les convictions religieuses qui sont faiblement citées entre 7 et 8 fois (Figure 2).

**Hésitation vaccinale face à la COVID-19:** les facteurs associés à l'acceptation, la résistance et l'hésitation vaccinale contre la COVID-19 en Afrique subsaharienne (SSA) varient selon plusieurs caractéristiques démographiques et sociales. L'âge, le niveau d'éducation, la région géographique et le statut professionnel jouent un rôle déterminant. Parmi les facteurs identifiés figurent la méfiance envers les industries pharmaceutiques, la qualité et la provenance des vaccins et aussi l'impact des médias et des réseaux sociaux [11,15-18]. De nombreux participants ont exprimé des inquiétudes quant à l'éthique et aux motivations des entreprises pharmaceutiques, craignant que les vaccins destinés à l'Afrique soient de moindre qualité [15,19-21].

Dans plusieurs études multicentriques menées en Afrique subsaharienne, les principaux facteurs influençant l'acceptation ou la réticence à la vaccination contre la COVID-19 incluent les préoccupations liées à la sécurité et aux effets secondaires des vaccins, qui constituent les raisons dominantes du refus [15,18,19,22-24]. Les ménages plus riches, les personnes plus instruites et les populations urbaines se sont révélés plus réticents à se faire vacciner, notamment en raison de ces inquiétudes. C'est le cas au Burkina Faso et au Malawi où les répondants urbains ont montré une plus grande hésitation comparée aux enquêtés ruraux [25-28]. En outre, la désinformation propagée sur les réseaux sociaux, notamment via des campagnes anti-vaccination, a intensifié la méfiance [11,22,29,30]. Le coût de la vaccination a également été identifié comme un facteur important, certains estimant qu'elle devrait être gratuite pour les populations les plus vulnérables [15].

**L'hésitation vaccinale face aux vaccins HPV:** l'hésitation vaccinale constitue un élément crucial dans la faible couverture du vaccin contre le papillomavirus humain (HPV) chez les adolescentes en Afrique en général [16,31-34]. Une étude menée par Khosa *et al*. en 2022 montre qu'en Afrique du Sud, plus de la moitié des filles ciblées par la vaccination contre l'HPV ne sont pas vaccinées. L'hésitation vaccinale, liée à des préoccupations concernant la sécurité du vaccin et ses effets secondaires, a été citée par 49,2% des parents d'enfants non vaccinés. Les parents des filles non vaccinées avaient des niveaux plus élevés de complaisance (perception d'un faible risque de la maladie) et de contraintes (manque d'accès à l'information), tandis que les parents dont les filles étaient vaccinées montraient plus de confiance dans le vaccin et un sens de responsabilité collective. De plus, l'impact de la désinformation propagée sur les réseaux sociaux, souvent provenant de pays à hauts revenus, a contribué à alimenter la méfiance contre le vaccin anti-HPV. Plusieurs études, au-delà de celle de Khosa *et al*. en 2022, révèlent également une méconnaissance des campagnes de vaccination contre l'HPV, soulignant d'importantes lacunes dans la communication. Ces résultats montrent la nécessité de renforcer les efforts d'éducation et de communication pour lutter contre la désinformation et améliorer l'acceptation vaccinale [16,34,35].

**Hésitation vaccinale concernant les vaccins du PEV:** on identifie trois catégories de facteurs: en premier les facteurs médicaux, en deuxième les facteurs socio-économiques et, enfin, les facteurs psychologiques. Les facteurs médicaux font intervenir les effets secondaires et l'historique vaccinal. Au titre des effets secondaires, relevons les craintes vis-à-vis des vaccins qui sont un facteur clé. Plus de 2/3 des participants aux études se disent préoccupés par les effets indésirables possibles, ce qui influence fortement l'hésitation et le refus de la vaccination. Concernant l'historique vaccinal, les personnes ayant déjà eu des effets secondaires graves ou mineurs sont plus enclines à hésiter ou à refuser la vaccination.

Les facteurs socio-économiques évoquent d'une part, l'âge, et d'autre part, le statut matrimonial et le nombre d'enfants. Ensuite, le niveau d'instruction et la confiance au gouvernement sont considérés. En effet, l'hésitation était plus forte chez les jeunes (18-29 ans). Comme le révèlent Kebe *et al*. dans une étude menée au Mali, le fait d'avoir moins de 30 ans s'est révélé être fortement associé à l'hésitation vaccinale [11]. Les jeunes adultes perçoivent un risque plus faible de la maladie, ce qui réduit leur intention de se faire vacciner. En outre, le fait d'avoir des enfants de moins de 5 ans et d'en avoir moins de 3 est aussi fortement lié à une hésitation vaccinale par rapport à la vaccination des enfants.

Les personnes n'ayant pas été scolarisées ou peu scolarisées sont plus enclines à accepter les messages négatifs des réseaux sociaux qui ont été indexés dans la diffusion des messages anti-vaccinaux. Le manque de confiance dans la gestion des épidémies et des pandémies par les gouvernements a augmenté la réticence vaccinale chez ceux ayant peu ou pas confiance dans les vaccins. S'agissant des facteurs psychologiques, les articles notent la mésinformation et la désinformation ainsi que l'influence religieuse et culturelle. La désinformation et les informations contradictoires sur la vaccination sont rapportées dans la majorité des études, entraînant la méfiance face aux vaccins. Pareillement, les croyances religieuses ou culturelles qui découragent la vaccination ont également été associées dans certaines études à des taux relativement faibles dans certaines régions. En ce qui concerne les pays africains, outre les croyances culturelles et religieuses, le recours à la médecine traditionnelle en première intention, les croyances à la théorie du complot (conspirations) selon laquelle les vaccins sont développés pour nuire aux Africains en les utilisant comme des cobayes sont des raisons supplémentaires enregistrées.

## Discussion

Les principaux facteurs d'hésitation vaccinale en Afrique subsaharienne incluent des dimensions médicales, socio-économiques et psychologiques. Sur le plan médical, les préoccupations inhérentes aux effets secondaires des vaccins jouent un rôle central, notamment pour les vaccins COVID-19 et HPV. De nombreux participants craignent des effets indésirables potentiels, et ceux ayant déjà eu des réactions vaccinales sont plus enclins à hésiter. Du côté socio-économique, les jeunes adultes (18-29 ans) et ceux à faible niveau d'instruction sont plus réticents à la vaccination, perçoivent un risque moindre de maladie et sont plus vulnérables aux messages anti-vaccinaux sur les réseaux sociaux. Le manque de confiance dans les gouvernements renforce cette réticence, en particulier dans la gestion des épidémies. Sur le plan psychologique, la désinformation, largement diffusée par les réseaux sociaux, nourrit la méfiance à l'égard des vaccins. De plus, les croyances religieuses et culturelles, combinées à des théories de conspiration et à la préférence pour les remèdes traditionnels, sont des facteurs clés d'hésitation vaccinale dans plusieurs pays africains.

Parmi les facteurs médicaux figurent la peur des effets secondaires, ainsi que les doutes quant à la sécurité et à l'innocuité des vaccins. Relativement à la sécurité vaccinale, notre étude a mis en évidence des doutes et craintes sur les vaccins dans plus de 80% des articles. Ce facteur associé à la réticence est aussi retrouvé dans des articles d'autres pays d'Afrique subsaharienne (Larson *et al*. en 2016) avec des taux variables par pays: RD Congo (13,8%), Afrique du Sud (9,8%), Nigeria (6,8%), Ghana (5,1%), Algérie (4,6%), Éthiopie (4,2%) [6]. Le taux de personnes remettant en question la sûreté des vaccins était globalement égale à 8% en Afrique de l'Ouest et 20% au Mali [11]. Ce scepticisme lié à la sécurité des vaccins est un facteur explicatif important de l'hésitation vaccinale. Ainsi au Mali, plus de 49% avaient déclaré avoir refusé un vaccin parce qu'ils le jugeaient « non sûr » ou parce qu'ils avaient « peur des effets indésirables », et 23% avaient déjà refusé ou hésité à vacciner un enfant car ils avaient émis des doutes sur la sécurité des vaccins et leur propension à entraîner des effets secondaires sur le long terme.

S'agissant des déterminants socio-économiques comme l'âge, le nombre d'enfants et le niveau de scolarisation, la revue suggère que le fait d'être âgé de moins de 30 ans, d'avoir un niveau d'éducation bas ou l'absence de scolarisation, moins de 3 enfants en bas âge étaient associés à l'hésitation vaccinale dans le contexte africain. Les jeunes adultes sont souvent plus hésitants en raison de leur perception d'un risque moindre de maladies évitables par la vaccination (36-38). En général, cette tranche d'âge tend à moins percevoir l'importance des vaccins, ce qui contribue à leur réticence. C'est une génération qui a très peu connu les grandes épidémies surtout celles évitables par la vaccination.

L'éducation joue un rôle complexe. Un niveau d'éducation élevé peut être associé à une plus grande acceptation des vaccins [11,36-39], mais dans certains cas, il est aussi lié à une plus grande réticence, surtout en raison d'une méfiance accrue envers les sources officielles et des préoccupations sur les effets secondaires. Ainsi, l'éducation influence l'hésitation vaccinale dans des directions parfois opposées. Le fait d'avoir plusieurs enfants, en particulier des jeunes, peut influencer l'hésitation vaccinale. Les parents ayant de jeunes enfants ou des familles nombreuses peuvent être plus prudents ou réticents vis-à-vis de la vaccination, en raison de la peur des effets secondaires sur leurs enfants.

La désinformation et la mésinformation, souvent véhiculées par les médias sociaux (TikTok, Facebook, WhatsApp), exacerbent les craintes liées aux vaccins, notamment en ce qui concerne leur sécurité et leurs effets secondaires [30,34,40,41]. Elles se réfèrent à une mauvaise compréhension ou à des erreurs non intentionnelles dans l'interprétation des informations scientifiques sur les vaccins. Cela inclut des malentendus sur la sécurité des vaccins, les effets secondaires et leur efficacité. La désinformation est souvent amplifiée par la circulation de données incorrectes dans les médias et sur les réseaux sociaux [34,40-43]. En conséquence, les personnes qui reçoivent des informations inexactes ou contradictoires peuvent développer des craintes injustifiées, augmentant ainsi leur résistance à la vaccination ou à la vaccination de leurs enfants.

Dans cette étude, plusieurs raisons motivent l'hésitation à se faire vacciner, les préoccupations relatives à l'innocuité et à l'efficacité des vaccins ainsi que la peur de la survenue des effets secondaires ont été retrouvées dans presque toutes les études. Ces préoccupations sont valables pour presque tous les vaccins disponibles sur le marché, en particulier les nouveaux vaccins (COVID-19 et HPV). Effectivement, Lehmann *et al*. en 2014 avaient démontré que les doutes sur l'efficacité des vaccins contre la grippe et ainsi la peur des effets indésirables constituaient les principaux motifs de rejet de la vaccination antigrippale [11,25,30,44]. Une situation comparable s'est produite en Sierra Leone lors de la vaccination contre l'Ebola relatée par Jalloh MF *et al*. Dans ce contexte, les préoccupations sécuritaires liées à un vaccin expérimental se sont révélées être le plus grand défi [23]. Un autre cas est celui des conclusions d'une revue systématique sur l'hésitation à la vaccination contre l'HPV en Europe qui avait relevé que la peur liée aux effets secondaires prédomine dans des recherches menées aux Pays-Bas, en Grèce, en Hongrie et en France tandis qu'en Espagne ce sont surtout les doutes sur l'efficacité du vaccin qui prédominent [24]. Nos résultats ont également mentionné la perception des risques.

**La perception du risque et les attitudes globales envers la vaccination:** plusieurs études ont également mis en évidence que le manque de confiance dans l'industrie pharmaceutique figure parmi les facteurs contribuant à l'hésitation vaccinale. Cette préoccupation soutient les conclusions d'une recherche effectuée par Singh [45] qui avait démontré que 58% des participants perçoivent les entreprises pharmaceutiques plus défavorablement que toute autre industrie.

Une désinformation par les réseaux sociaux a aussi été identifiée comme raison de réticence dans l'étude. Il est important de rappeler plusieurs affirmations sur la COVID-19 et une éventuelle vaccination les premiers jours de la maladie. Elles ont connu une généralisation et une expansion progressive au cours des années. Depuis mars 2020, diverses publications anti-vaccination sur le sol africain accusent les autorités scientifiques et politiques d'envahir les médias en ligne et les réseaux sociaux. Une des illustrations est la diffusion d'une émission du 1er avril 2020 sur la chaîne française d'information (LCI) impliquant des médecins français qui ont suggéré de mener des essais en Afrique [45]. Effectivement, l'assertion « L'Afrique sera-t-elle le terrain d'expérimentation pour les prochains vaccins contre le coronavirus? » s'est diffusée et a engendré un énorme émoi sur les réseaux sociaux [46]. Depuis ce moment, le vaccin est devenu un sujet majeur des « *fake news* » relatives à la COVID-19 sur le continent. Parmi ces fausses informations, on compte de fausses allégations concernant des campagnes de vaccination au Sénégal censées propager le virus, ainsi que des troubles anti-vaccination en Afrique du Sud ne se rapportant pas à cette question [46]. Cette situation a aussi provoqué la colère et l'indignation de nombreux artistes, tels que le footballeur ivoirien Didier Drogba ou l'association SOS Racisme [46] ainsi que des groupes antiracistes africains. De plus, l'étude des facteurs influençant l'hésitation vaccinale contre la COVID-19 dans trois régions du continent ciblé a relevé peu de différences. Toutefois, nous avons observé que certains facteurs étaient particulièrement plus mentionnés dans certaines régions.

Le continent africain a fait face à diverses croyances, qu'elles soient culturelles, religieuses ou conspirationnistes. L'analyse somalienne d'Ahmed *et al*. en 2021 faisait état de l'incorporation de matières étrangères non identifiées renfermant des dérivés de porc dans les vaccins [23], ou encore de la déclaration « je ne serai pas infectée, je crois en Dieu » rapportée au Nigéria [33]. De plus, des doutes sur la réalité même de la maladie ont vu le jour, le recours à la médecine traditionnelle a été évoqué ainsi que les croyances conspirationnistes avec des affirmations prétendant que les vaccins sont conçus pour nuire aux africains [33]. Dans cette étude, la plupart des personnes hésitantes ont affirmé que « le vaccin est destiné à tuer et stériliser les gens en Afrique » [11,39,45]. Des croyances sur l'expérimentation des vaccins en utilisant les africains comme des cobayes avaient également été enregistrés notamment, l'affirmation « les blancs profitent du système politique pour venir tester des vaccins et d'autres produits en Afrique qu'ils ne resteraient pas autrement dans leur propre pays et par conséquent, ils ne respecteront pas les procédures éthiques standard » ou encore « les africains n'ont pas besoin d'un vaccin COVID-19 puisqu'il existe des remèdes à base de plantes pour la maladie » ont été rapportés dans une étude camerounaise [16,43,47].

Ces observations suggèrent que, même si les déterminants sont presque semblables, il existe néanmoins une certaine variabilité en fonction du contexte et des réalités entre les régions du monde voire entre les pays de la même région. C'est ainsi qu'une prise en compte des spécificités s'impose en vue d'une meilleure planification des stratégies ciblées.

**Limites:** comme toute investigation scientifique, notre étude comporte quelques limites. D'abord, le fait de s'appuyer principalement sur PubMed comme source d'information pourrait avoir conduit à l'omission de certaines études pertinentes figurant dans les autres bases de données. En second lieu, l'utilisation de la littérature grise pour enrichir les recherches effectuées en Afrique pourrait avoir un impact sur la qualité et la crédibilité de certains résultats. Troisièmement, la majorité des études incluses sont transversales, fournissant une mesure de l'hésitation ou de l'acceptation vaccinale à un moment donné, ce qui peut ne pas refléter la réalité actuelle, étant donné la variabilité de l'hésitation vaccinale au fil du temps. Quatrièmement, la taille des échantillons varie considérablement d'une étude à l'autre, certaines étant plus robustes que d'autres, ce qui limite la comparabilité des résultats et nécessite une interprétation prudente. Cinquièmement, la collecte de données en ligne dans la majorité des études a restreint la participation des individus connectés et familiarisés avec les auto-évaluations en ligne. Enfin, la diversité des outils de collecte et d'analyse des données utilisés d'une étude à l'autre rend les méthodes d'évaluation de l'hésitation ou de l'acceptation vaccinale hétérogènes.

## Conclusion

L'hésitation vaccinale représente l'un des défis majeurs de la vaccination et de la santé publique. Cette revue systématique a permis de mettre en évidence les principaux déterminants de l'hésitation vaccinale en Afrique subsaharienne, avec des facteurs liés à la perception de la sécurité des vaccins, les croyances culturelles et religieuses, ainsi que l'influence significative de la désinformation propagée sur les réseaux sociaux. Ces éléments révèlent la complexité de l'hésitation vaccinale, particulièrement en ce qui concerne les vaccins contre la COVID-19 et l'HPV. Une approche ciblée prenant en compte les spécificités socioculturelles et une amélioration de la communication autour de la sécurité et des avantages des vaccins sont essentielles pour renforcer l'acceptation vaccinale et éviter les flambées de maladies évitables. Quelques particularités ont été retrouvées en Afrique subsaharienne, d'où sa complexité et la nécessité d'une prise en compte ciblée pour plus d'efficacité et d'efficience. L'analyse de l'hésitation vaccinale permettra également de mieux se préparer à la survenue d'éventuelles flambées épidémiques ou endémiques futures. C'est pourquoi des efforts de collaboration et de coordination entre les États, les partenaires, les décideurs en matière de santé, les communautés et les médias sont indispensables. Renforcer la confiance et l'acceptation des vaccins doit être au cœur du dialogue afin d'identifier et de mettre en œuvre des stratégies efficaces et durables pour le bien-être de tous.

### Etat des connaissances sur le sujet

- Phénomène présent et étudié dans beaucoup de pays d'Afrique au sud du Sahara;
- Une forte prévalence chez les agents de santé et les personnes ayant un niveau d'éducation bas;
- Il existe différents types d'hésitation vaccinale selon les vaccins.

### Contribution de notre étude à la connaissance

- Les différents facteurs associés à l'hésitation vaccinale;
- La similarité entre les pays d'Afrique au sud du Sahara;
- La présence d'hésitation spécifique notamment pour le vaccin de l'HPV, la COVID-19.

## Remerciements

Nos remerciements à l'université Senghor à Alexandrie, en Égypte à travers le département de santé et l'école doctorale interdisciplinaire en développement africain. Nous remercions également le programme international porté par l’Académie internationale de la francophonie scientifique de l’AUF, dont Amadou Tila Kebe est lauréat dans le cadre de l’Université d’été de la francophonie (UDEF), dédié à la promotion de la publication scientifique francophone et plurilingue. Ce dispositif lui a permis de bénéficier d’un accompagnement d’excellence, comprenant un mentorat scientifique, des évaluations en double aveugle ainsi que des ateliers de haut niveau consacrés à la publication, à l’édition, à la diffusion et à la valorisation de la recherche.
